# Dyspnea in Patients Receiving Radical Radiotherapy for Non-Small Cell Lung Cancer: A Prospective Study

**DOI:** 10.3389/fonc.2020.594590

**Published:** 2020-12-23

**Authors:** Angela Sardaro, Fiona McDonald, Lilia Bardoscia, Konstantin Lavrenkov, Shalini Singh, Sue Ashley, Daphne Traish, Cristina Ferrari, Icro Meattini, Artor Niccoli Asabella, Michael Brada

**Affiliations:** ^1^ Lung Research Unit, The Royal Marsden NHS Foundation Trust, Sutton, United Kingdom; ^2^ Interdisciplinary Department of Medicine, Nuclear Medicine Unit and Section of Radiology and Radiation Oncology, University of Bari Aldo Moro, Bari, Italy; ^3^ Academic Radiotherapy Unit, The Institute of Cancer Research, Sutton, United Kingdom; ^4^ Radiation Therapy Unit, Department of Oncology and Advanced Technology, Azienda USL-IRCCS di Reggio Emilia, Reggio Emilia, Italy; ^5^ Department of Oncology, Soroka University Medical Center, Faculty of Health Sciences, Ben-Gurion University of the Negev, Beer Sheva, Israel; ^6^ Sanjay Gandhi Post Graduate Institute of Medical Sciences (SGPGIMS), Department of Radiotherapy, Lucknow, India; ^7^ Department of Biomedical, Experimental, and Clinical Sciences, University of Florence, Radiation Oncology Unit - Oncology Department, Azienda Ospedaliero-Universitaria Careggi, Florence, Italy; ^8^ Department of Radiation Oncology, University of Liverpool and Clatterbridge Cancer Centre NHS Foundation Trust, Wirral, United Kingdom

**Keywords:** non-small cell lung cancer, radiotherapy, dyspnea, dose-volume parameters, radiation-induced lung injury

## Abstract

**Background and Purpose:**

Dyspnea is an important symptomatic endpoint for assessment of radiation-induced lung injury (RILI) following radical radiotherapy in locally advanced disease, which remains the mainstay of treatment at the time of significant advances in therapy including combination treatments with immunotherapy and chemotherapy and the use of local ablative radiotherapy techniques. We investigated the relationship between dose-volume parameters and subjective changes in dyspnea as a measure of RILI and the relationship to spirometry.

**Material and Methods:**

Eighty patients receiving radical radiotherapy for non-small cell lung cancer were prospectively assessed for dyspnea using two patient-completed tools: EORTC QLQ-LC13 dyspnea quality of life assessment and dyspnea visual analogue scale (VAS). Global quality of life, spirometry and radiation pneumonitis grade were also assessed. Comparisons were made with lung dose-volume parameters.

**Results:**

The median survival of the cohort was 26 months. In the evaluable group of 59 patients there were positive correlations between lung dose-volume parameters and a change in dyspnea quality of life scale at 3 months (V_30_ p=0.017; V_40_ p=0.026; V_50_ p=0.049; mean lung dose p=0.05), and a change in dyspnea VAS at 6 months (V_30_ p=0.05; V_40_ p=0.026; V_50_ p=0.028) after radiotherapy. Lung dose-volume parameters predicted a 10% increase in dyspnea quality of life score at 3 months (V_40_; p=0.041, V_50_; p=0.037) and dyspnea VAS score at 6 months (V_40_; p=0.027) post-treatment.

**Conclusions:**

Worsening of dyspnea is an important symptom of RILI. We demonstrate a relationship between lung dose-volume parameters and a 10% worsening of subjective dyspnea scores. Our findings support the use of subjective dyspnea tools in future studies on radiation-induced lung toxicity, particularly at doses below conventional lung radiation tolerance limits.

## Introduction

Radical radiotherapy (RT), with or without chemotherapy has an established role as an alternative to surgery in medically inoperable, localized and locally advanced non-small cell lung cancer (NSCLC) (1, 2). In particular, chemoradiation represents the standard of care for locally advanced disease (3–5). The disappointing survival rates following radical conventionally fractionated RT have been the impetus behind application of advanced RT techniques with the aim of increasing radiation dose intensity without additional toxicity (6–9).

Radiation-induced lung injury (RILI) remains a significant limiting factor to dose escalation. Knowledge of the effect of radiation on lung is imperative for optimization and comparison of the relative merits of different RT plans. The risk of radiation pneumonitis (RP), an interstitial pulmonary inflammatory process usually developing within 6 months of RT, is the predominant endpoint used to quantify RILI, forming the basis of recommended RT dose-volume constraints obtained by lung dose volume histogram (DVH) in conventional RT (10). However, the grading of RP is challenging as the most frequently used scoring systems, including the Common Terminology Criteria for Adverse Events (CTCAE) and the Radiation Therapy Oncology Group (RTOG) system, have a small number of broad categories combining symptomatic, functional, and radiological criteria in addition to indication of medical intervention. In addition, the incidence of clinically significant RP is low and therefore, it is not discriminatory at doses below conventional tolerance defined by incidence of RP.

Arguably, the most clinically relevant endpoint for patients is the worsening of symptoms, particularly dyspnea. A more discriminating measure of the effect of radiation on dyspnea may be useful for weighing up the potential risks and benefits of a RT plan at doses below conventional tolerance defined by the incidence of RP. We carried out an explorative, prospective assessment of dyspnea based on the hypothesis that RILI below conventional tolerance may be detected and quantifiable where dyspnea assessment may offer a more discriminatory and objective measure.

## Materials and Methods

### Patient Population

Between February 2003 and January 2011, patients were invited to participate in a prospective observational study following approval by the institution’s Committee for Clinical Research and Local Research Ethics Committee. The trial was conducted in accordance with European Union guidelines for Good Clinical Practice and signed informed consent was obtained from participants. All patients scheduled to receive radical RT to a dose of 64 Gray (Gy) in 32 daily fractions were eligible for study entry if they fulfilled the following criteria: histological or radiological diagnosis of localized medically inoperable or unresectable locally advanced NSCLC (AJCC 6^th^ edition stages I-III, excluding T4 lesions associated with pleural effusion), baseline forced expiratory volume in 1 s (FEV1) greater than 40% of predicted normal value and World Health Organization (WHO) performance status 0-2.

### Radiotherapy Planning and Delivery

A planning helical computed tomography (CT) scan of the thorax was acquired with the patient positioned on a chest board either in free breathing or breath-hold using the active breathing control (ABC) device with 2.5–3 mm slice thickness (11, 12). RT planning was performed using the Pinnacle^3^ planning software (Philips Medical Systems Madison, WI). The extent of the gross tumor volume (GTV) was defined using CT lung windows (Width=1600, Length=-300) with reference to diagnostic imaging. The clinical target volume (CTV) was considered the same as the GTV. In patients treated in free breathing, a margin of 1.5 cm was added cranio-caudally with axially 1 cm for central disease and 1.5 cm for peripheral disease added to the CTV to create the planning target volume (PTV). In patients treated with ABC, an isotropic margin of 1 cm was added from CTV to PTV. Conformal plans were created to ensure adequate coverage of the PTV in accordance with International Commission on Radiation Units (IRCU) 50 and 62 recommendations, whilst maintaining the constraints for organs at risk. Treatment was delivered in a single phase to a dose of 64 Gy in 32 daily fractions prescribed to the 100% isocenter using a linear accelerator (Elekta, Crawley, UK).

### Radiotherapy Lung DVH Parameters

Both lungs were considered together as a single paired organ and contoured on the planning scan using CT lung windows. Care was taken to ensure inclusion of the whole lung tissue from apices to bases including regions of collapse or consolidation. The extent of the GTV/CTV, trachea and proximal bronchial tree were excluded from the volume. The total mean lung dose (MLD) was recorded for each patient in addition to the percentage of the total lung volume at threshold doses of radiation in Gy (V_dose_) ranging from 20 Gy to 60 Gy in 10 Gy increments (V_20_, V_30,_ V_40,_ V_50,_ V_60_). All plans met the dose constraints of a V_20_ ≤30% and a MLD of ≤18 Gy.

### Pre-Treatment and Follow-Up Assessments and Dyspnea Scales

Dyspnea, pneumonitis, spirometry and quality of life (QoL) were prospectively assessed at baseline prior to treatment, at 3, 6, 9, and 12 months after completion of RT and then 6 monthly until disease progression or death. Patients were imaged with chest radiograph or CT scan at follow-up time-points. At baseline patients were asked to complete the Adult Comorbidity Evaluation 27 questionnaire (ACE-27) (13). At each scheduled study appointment patients were assessed clinically and the physician-scored pneumonitis grade (CTCAE) was recorded (14). Patients were asked to complete the European Organisation for Research and Treatment of Cancer (EORTC) QoL questionnaire including the lung module (QLQ-LC13) (15, 16). Dyspnea was assessed using the breathlessness section of QLQ-LC13 and from patients’ marking the dyspnea visual analogue scale (VAS), a 100 mm long vertical line, to indicate their degree of breathlessness (17). Each VAS was separately recorded without reference to previous reading. Pulmonary function tests (PFT) consisted in FEV1 and forced vital capacity (FVC) measured using an Alpha III spirometer (Vitalograph, Lenexa, KS) and were recorded as the percentage of the predicted value. Ventilation parameters were chosen for correlative analysis because strictly representative for respiratory function and capacity, unlike perfusion parameters possibly affected by confounding factors, such as cardiac and/or hematological comorbidities.

### Statistical Analysis

A sample size of at least 30 lung cancer patients was arbitrary defined, since this was an explorative, prospective study and no similar study designs to compare with for accrual evaluation have ever been reported in literature. Statistical analysis will eventually be descriptive for future findings and data integration.

Survival analysis from the start of radiation treatment was performed using the Kaplan-Meier method. As the primary objective was to assess changes in dyspnea and other measures of lung function due to radiation, patients with progressive disease were censored for dyspnea assessment at the time of disease progression. Median follow-up, progression free survival (PFS) and overall survival (OS) were calculated with 95% confidence intervals (CI).

Data were taken from the 3 QoL questions related to breathlessness (**Table 1**) and the calculated dyspnea QoL score was normalized to a 100 point scale (16). The dyspnea VAS was assessed and attributed a score from 0 to 10 to the nearest millimeter. The global QoL score was calculated from 0 to 100. The pneumonitis grade and the percentage of predicted normal values for FEV_1_ and FVC were documented.

**Table 1:** EORTC QLQ-LC13 dyspnea QoL assessment.

During the past week:	Not at All	A Little	Quite a Bit	Very Much
Were you short of breath when you rested?	1	2	3	4
Were you short of breath when you walked?	1	2	3	4
Were you short of breath when you climbed stairs?	1	2	3	4

Changes in dyspnea QoL, dyspnea VAS, global QoL, FEV1, and FVC from the baseline pre-RT measurement were detected for individual patients at each post-irradiation time-point. A positive change indicated a worsening of dyspnea QoL, dyspnea VAS and global QoL and an improvement in FEV1 and FVC. Comparisons between the mean changes and the corresponding baseline values for the cohort were performed with 95% CI at each post-RT time-point and correlations with lung DVH parameters at 3, 6, and 12 months post-RT were assessed using rank correlation coefficients. The association between the rate of ≥ grade 2 RP at 3 months after RT and lung DVH parameters was calculated using a rank correlation coefficient. The rate of RP at other time-points was considered too much low for further correlation assessments.

Where a significant correlation at the 5% level was observed between lung DVH parameters and changes from baseline post-RT, the Mann Whitney test was performed to test for correlation of lung DVH parameters with a clinically relevant worsening of dyspnea or pulmonary function. For the purposes of statistical analysis, a clinically relevant worsening was defined as follows: 10% increase in dyspnea QoL compared to baseline, 10% increase in dyspnea VAS compared to baseline, and 10% decrease in FEV1 or FVC compared to baseline. Exploratory receiver operator curve (ROC) analyses were also carried out to assess for an optimal cut-off to predict worsening of dyspnea or pulmonary function following treatment.

## Results

### Patient Population, Follow-Up and Disease Outcome

Eighty consecutive patients during the study period fitted the selection criteria and accepted to participate to the study. Among these, 21 patients were excluded from further analysis: five had missing pre-RT dyspnea assessment, one did not complete RT due to pulmonary embolism, eight had missing 3-month post-RT dyspnea assessment and seven developed disease progression prior to 3-month post-RT assessment. Data from the remaining 59 patients were analyzed for the study purpose. The characteristics of the population in study are summarized in **Table 2**. In particular, 34 (57.6%) patients suffered from cardiac and/or hematological comorbidities, and 54 (91.5%) of them reported smoke habit.

**Table 2:** Patient and disease characteristics.

Patient characteristics	N = 59	%	Mean (SD)
**Gender**			
Male	35	59	
Female	24	41	
**Age in years**			69 (10)
**Performance status (WHO)**			
0	19	32	
1	38	64	
2	2	4	
**Co-morbidity score**			
0	13	22	
1	15	26	
2	17	29	
3	12	20	
Missing	2	3	
**Smoking status**			
Current	54	92	
Never smoker or ex-smoker	5	8	
**Disease characteristics**			
**Histological diagnosis**			
Squamous cell carcinoma	24	41	
Adenocarcinoma	14	24	
Other	5	8	
Missing	16	27	
**Disease stage (AJCC 6^th^ ed)**			
I	14	24	
II	7	12	
IIIA	19	32	
IIIB	18	30	
**Neoadjuvant chemotherapy**	24	41	
**Prior lobectomy**	3	5	

WHO, World Health Organization; AJCC, American Joint Committee on Cancer.

**Figure 1:**
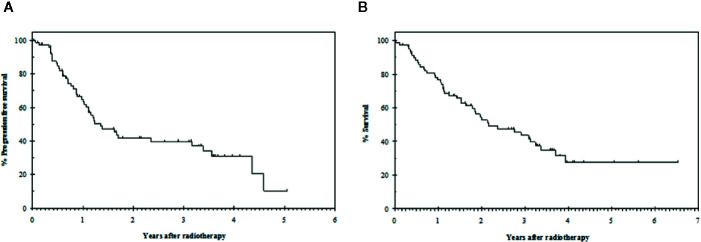
**(A)** PFS and **(B)** OS in a cohort of 80 patients treated with radical RT.

With a median follow-up of 20 months (range 0 to 78), the median progression free survival was 16 months (95% CI: 10–23) and the median overall survival was 26 months (95% CI: 14–38) (**Figure 1**).

### Baseline Measurements and Compliance

The mean baseline dyspnea QoL, dyspnea VAS, global QoL, FEV1 and FVC and lung dose-volume data for the cohort are summarized in **Table 3**. All 59 patients had clinical assessments at baseline and 3 months post-RT. Taking withdrawal of patients from further follow-up due to disease progression and death into account, 2/48 (4%), 16/39 (41%), 1/35 (3%) had missing follow-up assessments at 6, 9, and 12 months, respectively, with no missing assessments but few surviving patients at 18 months post-RT excluded from further analysis, data attributable to the prognosis of the percentage of patients with IIIA and IIIB disease stage.

**Table 3:** Baseline assessment and normal lung DVH data.

Measurement	Mean	95% CI
Dyspnea QoL (n=59)	26	21–31
Dyspnea VAS (n=59)	2.2	1.5–2.8
Global QoL (n=58)	67	63–72
FEV1% of predicted (n=56)	69	64–74
FVC % of predicted (n=55)	86	80–91
MLD (Gy) (n=59)	12	11–13
V20 (%) (n=59)	23	20–26
V30 (%) (n=59)	18	15–21
V40 (%) (n=59)	13	11–16
V50 (%) (n=59)	9	7–12
V60 (%) (n=59)	6	4–9

QoL, quality of life; VAS, visual analogue scale; FEV1, forced expiratory volume in 1 s; FVC, forced vital capacity; MLD, mean lung dose; V_dose_, percentage of the total lung volume at threshold doses of radiation in Gy.

### Change in Dyspnea Quality of Life, Dyspnea VAS, Global Quality of Life, FEV 1 and FVC from Baseline, and Rate of Radiation Pneumonitis

**Figure 2:**
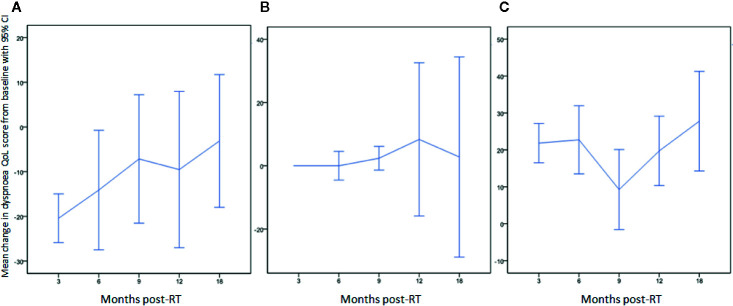
Change in dyspnea QoL from baseline at different time-points by classifying patients as those who **(A)** initially improved (QoL score decreased: 20/59; 34%), **(B)** remained stable (10/59; 17%), or **(C)** initially worsened (QoL score increased: 29/59; 49%) between baseline and 3 months post-RT.

The mean dyspnea QoL score of the cohort increased by 4 (95% CI: -2–10) at 3 months after irradiation. Twenty-nine patients (49%) had worse dyspnea QoL with a mean increase in score of 22 (95% CI: 17–27); 20 patients (34%) had improved dyspnea QoL with a mean decrease in score of 20 (95% CI: 15–26) and 10 patients (17%) had no change in QoL score. The mean change from baseline at follow-up time-points is displayed in **Table 4**. Changes in dyspnea QoL from baseline at different time-points by classifying patients as those who initially improved, remained stable, or worsened between baseline and 3 months post-RT are displayed in **Figure 2**. The mean change in dyspnea VAS, global QoL, FEV1, and FVC from baseline at the follow up time-points is reported in **Table 4**. At 3 months post-RT eight (14%), two (3%), and two (3%) patients had grade 1, grade 2, and grade 3 RP, respectively. At 6 months after the treatment, three (7%), one (3%), and one (3%) patients had grade 1, grade 2, and grade 3 RP, respectively.

**Table 4:** Mean change from baseline of dyspnea QoL, dyspnea VAS, global QoL, and percentage of predicted FEV1 and FVC at time-points post-RT with 95% CI in parentheses.

Time post-RT (months)	Mean change in % dyspnea scores (range)				
	3	6	9	12	18
Dyspnea QoL score	4 (-2 to 10)	7 (0 to15)	2 (-4 to 8)	11 (3 to 20)	15 (5 to 26)
Dyspnea VAS score	1.0 (0.2 to 1.8)	1.7 (0.8 to 2.7)	1.3 (0.3 to 2.3)	1.7 (0.5 to 2.8)	2.1 (0.8 to 3.5)
Global QoL score	1 (-5 to 7)	-6 (-14 to 3)	-10 (-21 to 2)	-8 (-16 to 0)	-5 (-16 to 6)
% of predicted FEV1	2 (-1 to 4)	1 (-3 to 6)	2 (-5 to 8)	0 (-5 to 6)	2 (-4 to 8)
% of predicted FVC	2 (-3 to 8)	-2 (-6 to 2)	3 (-7 to 12)	-1 (-7 to 6)	2 (-6 to 9)

### Relationship Between Lung DVH and Measures of Lung Function

**Table 5** shows the relationship between dyspnea and lung function measures including dyspnea QoL, dyspnea VAS, FEV1, FVC, and incidence of RP. Change in dyspnea QoL score at 3 months correlated with the lung V_30_, V_40_, V_50_, and MLD (p=0.017, p=0.026, p=0.049, and p=0.05, respectively). There was no significant correlation between lung DVH parameters and change in dyspnea VAS, global QoL, FEV1, FVC, and rate of ≥ grade 2 RP at 3 months. Change in dyspnea VAS score at 6 months correlated with the lung V_30_, V_40_, and V_50_ (p=0.05, p=0.026 and p=0.028, respectively). No significant correlation between lung DVH parameters and change in dyspnea QoL, global QoL, FEV1 or FVC was demonstrated at 6 months. At 12 months there was a significant negative correlation between the change in FVC and the lung V_40_ and V_60_ (p=0.043 and p=0.046, respectively) and between the change in FEV1 and lung V_40_, V_50_, and V_60_ (p=0.016, p=0.011, and p=0.005, respectively).

**Table 5:** Rank correlation between normal lung DVH parameters and lung function.

DVH Parameter Correlations		Change in Dyspnea QoL			Change in Dyspnea VAS			Change in Global QoL			Change in FVCas % of predicted			Change in FEV1as % of predicted			Rate of ≥ grade 2 RP
		3m	6m	12m	3m	6m	12m	3m	6m	12m	3m	6m	12m	3m	6m	12m	3m
N		59	44	30	59	45	33	57	44	32	52	38	29	52	39	29	4
V20	Co	0.22	0.15	0.00	0.18	0.18	0.17	-0.12	-0.06	0.27	-0.09	-0.23	-0.24	-0.05	-0.15	-0.15	0.45
	Sig	0.088	0.340	0.995	0.180	0.247	0.335	0.378	0.688	0.139	0.540	0.169	0.205	0.745	0.373	0.432	0.553
V30	Co	0.31	0.27	0.19	0.21	0.29	0.22	-0.20	-0.15	-0.03	-0.06	-0.23	-0.31	-0.08	-0.19	-0.29	0.89
	Sig	**0.017**	0.074	0.327	0.107	**0.050**	0.220	0.145	0.336	0.879	0.659	0.172	0.097	0.585	0.247	0.129	0.106
V40	Co	0.29	0.20	0.18	0.17	0.33	0.31	-0.17	-0.12	-0.05	0.00	-0.13	-0.38	-0.85	-0.17	-0.45	0.89
	Sig	**0.026**	0.184	0.351	0.207	**0.026**	0.080	0.194	0.438	0.798	0.979	0.442	**0.043**	0.549	0.309	**0.016**	0.106
V50	Co	0.26	0.232	0.12	0.13	0.33	0.22	-0.17	-0.10	0.05	0.06	-0.14	-0.35	-0.05	-0.22	-0.47	0.45
	Sig	**0.049**	0.13	0.539	0.335	**0.028**	0.229	0.217	0.530	0.807	0.669	0.400	0.062	0.728	0.185	**0.011**	0.553
V60	Co	0.13	0.15	-0.02	0.11	0.28	0.17	-0.08	0.02	0.14	0.13	-0.08	-0.37	-0.04	-0.27	-0.50	0.00
	Sig	0.327	0.333	0.915	0.402	0.060	0.348	0.549	0.879	0.452	0.346	0.625	**0.046**	0.799	0.099	**0.005**	1.000
MLD	Co	0.26	0.06	-0.22	0.05	0.11	-0.03	-0.14	-0.01	0.16	-0.14	-0.20	-0.34	-0.22	-0.09	-0.23	0.45
	Sig	**0.050**	0.688	0.240	0.717	0.476	0.858	0.285	0.939	0.396	0.342	0.234	0.069	0.123	0.590	0.222	0.553

m, months; N, number of patients; Co, correlation coefficient; Sig, 2-tailed significance.

Significant correlations shown in bold.

### ROC Analysis for Lung Damage Post-Radiotherapy

**Figure 3:**
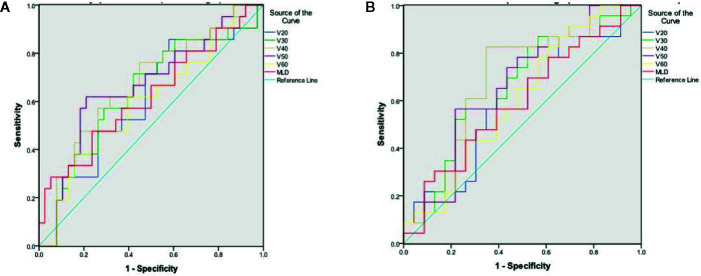
Receiver Operator Curve **(A)** for 10-point change in dyspnea QoL between baseline and 3 months post-RT **(B)** for 1-point change in dyspnea VAS between baseline and 6 months post-RT.

Lung damage defined by a 10% increase in dyspnea QoL score at 3 months correlated with the lung V_40_ and V_50_ with an area under the curve (AUC) of 0.66 (p=0.041) and 0.66 (p=0.037), respectively (**Figure 3**). Lung damage defined by a 10% increase in dyspnea VAS score at 6 months correlated to the lung V_40_ with an AUC of 0.69 (p=0.027) (**Figure 3**). A cut off of 11% for the V_40_ was associated with a sensitivity of 76% and a specificity of 53% for predicting worsening of dyspnea by a 10% increase in dyspnea QoL score at 3 months, and a sensitivity of 83% and a specificity of 61% by a 10% increase in dyspnea VAS score at 6 months post-RT. ROC analysis demonstrated that no DVH parameter significantly predicted clinically relevant lung damage defined by a 10% decrease in FVC or FEV1 (% of predicted) at 12 months post-RT compared to baseline.

## Discussion

Worsening of dyspnea is a characteristic feature of clinically relevant RILI (18). Our study aimed to prospectively evaluate subjective dyspnea changes following radical RT for NSCLC as a measure of potential lung toxicity when treating to disease below radiation tolerance of the lung.

The study demonstrated a significant correlation between lung DVH parameters and change in dyspnea post-RT using two different patient-completed dyspnea tools. Three months post-RT, a change in dyspnea QoL score significantly correlated with lung DVH parameters (V_30_ p=0.017; V_40_ p=0.026; V_50_ p=0.049; MLD p=0.05). Six months post-RT, a change in dyspnea VAS score significantly correlated with lung DVH parameters (V_30_ p=0.05; V_40_ p=0.026; V_50_ p=0.028). Lung DVH parameters were significantly predictive for a 10% increase in dyspnea QoL score 3 months post-RT (V_40_; p=0.041, V_50_; p=0.037) and dyspnea VAS score 6 months after the treatment (V_40_; p=0.027), respectively.

The observed rate of ≥ grade 2 RP at 3 and 6 months post-RT was low (6%) with no correlation observed between rate of ≥ grade 2 RP and lung DVH parameters. This low rate of RP is to be expected given that the lung dose-volume constraints were met for all RT plans. Despite a significant negative correlation at 12 months between any change in the percentage of predicted FVC and lung V_40_ and V_60_, respectively, and between any change in percentage of predicted FEV1 and lung V_40_, V_50_, and V_60_, respectively, no lung DVH parameters were significant predictors of a clinically relevant worsening of FVC or FEV1, defined in this study by a 10% reduction in percentage of predicted values.

Dyspnea in patients with NSCLC is multi-factorial and is affected by respiratory and cardiac comorbidity (18, 19). While dyspnea is the predominant symptom in classical RP, the clinical diagnosis of RP is challenging due to confounding cardio-respiratory conditions affecting the lung cancer patient population (20). In addition, baseline respiratory function/dyspnea can be an additional risk factor for RILI (21).

Dyspnea is a subjective symptom not easily validated with objective tests. Nevertheless, it is of primary importance to the patient and in the absence of RP may provide a more sensitive measure of small changes to lung function and arguably a more appropriate measure for monitoring the patterns and severity of dyspnea over time. This study explored the relationship between lung dose-volume information and patient-recorded changes in dyspnea following irradiation. Measurement of relative dyspnea compared to baseline pre-RT values was performed to account for comorbidities as a confounding factor. However, given the complexity of dyspnea as a symptom, two tools were used to permit both a unidimensional dyspnea assessment with the VAS (17) and a lung cancer specific dyspnea assessment tool derived from the EORTC QOL questionnaire (15, 16). Such an approach to assessment of dyspnea had been suggested in a systematic review of the available tools (22) and a 10% change from baseline values is a reasonable measure of a clinically meaningful change (23). The dyspnea scales used in this study were demonstrated as a valid and reliable tool in a range of cancer patient populations, including lung cancer patients, and confirmed to reflect the common symptoms and treatment-related toxicities underlying radio(chemo)therapy (24). Another limitation of the study is the multiple testing in a small number of patients and the associated increased potential for Type I error in the results. Therefore, our results require validation in a larger cohort of patients.

Advances in planning software and delivery techniques permitted increasing flexibility when adjusting RT plans to spare normal tissue while maintaining target coverage. Distilling lung 3-dimensional dose-volume distribution data down to a threshold metric for risk of RILI produce a range of thresholds for various metrics in the RP literature (25–38). This is likely to be due to the gradual increase in lung damage with radiation dose (18). However, recommended thresholds for MLD and lung V_20_ with conventional fractionation remain widely used as normal tissue dose-constraints and are considered useful to aid assessment and optimization of different RT plans (10, 39). In this series, we recorded MLD 11% to 13% Gy and V_20_ 20% to 26%, in line with the accepted thresholds to minimize the risk of RILI (40). While the relatively small numbers in this study limited the statistical power of the results, ROC curve analyses suggested that the percentage volume of lung receiving 40 Gy (V_40_) may be predictive for an increase in subjective dyspnea following conventionally fractionated RT. A lung V_40_ threshold of 11% may be a useful additional constraint and warrants validation in a larger cohort of patients.

We report the first radiotherapy study to describe the relationship between lung DVH parameters and self-assessed dyspnea scores. There have been studies of physician scored dyspnea which is recognized to suffer from investigator bias. Lung DVH parameters have shown no correlation with a change in physician-scored dyspnea score in stage I NSCLC patients receiving stereotactic RT (41). The evolution of dyspnea following radical RT for stage I-III NSCLC has also been studied in 197 patients using the physician-scored CTCAE classification (dyspnea grades 0–4) with worsening dyspnea in 17% to 27% of patients. The investigators highlighted the need for assessing dyspnea at more than one time-point post-RT (42).

To date, radiobiological parameters, rather than subjective dyspnea tools, have gained increasing interest in preventing RILI for thoracic irradiation. Recent publications argue in favor of NTCP model as a possible way to optimize treatment plans according to the probability of RP (18), and a multinomial NTCP has been proposed as possibly predictive for dyspnea grade with high accuracy (43). The need for intensification of local treatment to achieve better local control and improve survival rates for NSCLC without additional toxicity has also given rise to several, promising, dose escalation studies in United Kingdom, based on prespecified and mean lung dose constraints to increase tumor control probability without worsening normal tissue complication probability (NTCP) (44–49).

Radiotherapy dose-independent clinical factors impact on the risk of RILI (18, 50) and include age and comorbidity (50), smoking status (51), tumor location (52), systemic therapy (53, 54) and target therapies (18). The risk of RILI can also be affected by dose-dependent factors related to the irradiation of the heart rather than lung (55). Development of multi-factorial models including clinical and dosimetric factors for prediction of risk of RILI is important. Such a model was developed using a physician assessed dyspnea score (CTCAE version 3.0 (14)) as the endpoint (56). Addition of clinical factors to dosimetric factors improved the performance of the model in predicting for severe dyspnea post-RT. The use of patient-scored dyspnea assessments may further improve the performance of such models. However, these are only appropriate at doses close to or beyond conventional accepted tolerance limits and do not provide information on the effect of radiation at doses below tolerance limits.

In conclusion, dyspnea is a prominent symptom of RILI, which remains an important limitation for radical treatment of NSCLC with RT. Monitoring changes in dyspnea as an endpoint for multi-factorial predictive models of lung toxicity is important to increase the efficacy of radio(chemo)therapy without compromising treatment safety. Given the subjective nature of the symptom, patient-completed tools may be more sensitive and subject to less bias than physician grading. We have demonstrated that lung dose-volume parameters predict for a 10% worsening of dyspnea QoL at 3 months and dyspnea VAS at 6 months post-RT. A constraint of 11% of the lung volume receiving 40 Gy, if validated, may be useful in limiting the proportion of patients who experience ≥10% increase in dyspnea score following conventional RT. Further estimates, including competing risk analysis, will be needed to define the complex relationship among dyspnea, lung cancer and RILI in detail, also taking into account the rate of locally advanced disease stage. Our findings support the use of subjective dyspnea tools in future studies on lung RT toxicity.

## Data Availability Statement

The raw data supporting the conclusions of this article will be made available by the authors only after the authorization of the Study Coordinator (MB). 

## Author Contributions

FM, DT and AS collected and managed the data, and wrote the manuscript together with MB. FM developed the project, analyzed the data together with SA, and edited the manuscript. LB reviewed the literature and edited the manuscript. KL and SS interpreted the data. CF edited the manuscript. IM collected, managed, and analyzed the data, and wrote the manuscript. AA edited the manuscript. MB developed the project, peformed data integrity check and data analysis accuracy check, and edited the manuscript. All authors contributed to the article and approved the submitted version.

## Conflict of Interest

The authors declare that the research was conducted in the absence of any commercial or financial relationships that could be construed as a potential conflict of interest.
